# Influenza vaccination of pregnant women protects them over two consecutive influenza seasons in a randomized controlled trial

**DOI:** 10.1080/14760584.2016.1192473

**Published:** 2016-06-06

**Authors:** Eleonora Mutsaerts, Shabir A. Madhi, Clare L. Cutland, Stephanie Jones, Andrea Hugo, Siobhan Trenor, Florette K. Treurnicht, Kerstin Klipstein-Grobusch, Adriana Weinberg, Marta C. Nunes

**Affiliations:** ^a^ Department of Science and Technology/National Research Foundation, Vaccine Preventable Diseases, University of the Witwatersrand, Johannesburg, South Africa; ^b^ Respiratory and Meningeal Pathogens Research Unit, Medical Research Council, University of the Witwatersrand, Johannesburg, South Africa; ^c^ Julius Global Health, Julius Center for Health Sciences and Primary Care, University Medical Center Utrecht, Utrecht, The Netherlands; ^d^ National Institute for Communicable Diseases, Division of National Health Laboratory Service, Centre for Respiratory Diseases and Meningitis, Johannesburg, South Africa; ^e^ Division of Epidemiology and Biostatistics, School of Public Health, Faculty of Health Sciences, University of the Witwatersrand, Johannesburg, South Africa; ^f^ Department of Pediatrics, Medicine and Pathology, University of Colorado, Aurora, CO, USA

**Keywords:** Influenza vaccine, efficacy, immunogenicity, maternal immunization

## Abstract

**Background**: We assessed the persistence of hemagglutinin inhibition (HAI) antibodies and the vaccine efficacy (VE) of trivalent inactivated influenza vaccine (IIV3) following vaccination of a cohort of pregnant South African women during a second influenza season.

**Methods**: A cohort of women who participated in a randomized placebo-controlled trial on the safety, immunogenicity and efficacy of IIV3 in 2011 had HAI titers measured in 2012 and were monitored for influenza illness until the end of 2012.

**Results**: The proportion of women with HAI titers ≥1:40 was significantly greater in vaccinees (63%) compared to placebo-recipients (22%; p < 0.001). VE in 2012 was 63.8% (95% confidence interval [95%CI]: −33.7%, 90.2%); combined VE for 2011 and 2012 was 58.3% (95%CI: 0.2%, 82.6%).

**Conclusion**: The majority of women who received IIV3 during pregnancy had HAI titers above the putative threshold for protection against influenza illness one year after vaccination and showed a trend towards protection against influenza disease.

## 1. Introduction

Seasonal influenza virus is responsible for three to five million cases of severe influenza disease worldwide and 250,000–500,000 annual deaths [1]. Pregnant women are at increased risk of severe influenza illness and death compared with nonpregnant adults [2]. Influenza illness during pregnancy has also been associated with increased rates of stillbirths, neonatal deaths, preterm deliveries and reduced birth weight [3–6]. Data from South Africa showed that pregnant women had a threefold increased risk of death associated with seasonal and 2009 pandemic (A/H1N1pdm09) influenza compared to nonpregnant women, particularly if HIV-infected [7,8].

We previously reported that trivalent inactivated influenza vaccine (IIV3) was safe and 50% efficacious in HIV-uninfected pregnant women and 49% efficacious in their infants until 24 weeks postpartum against polymerase-chain reaction-confirmed influenza (PCR-CI) illness [9]. Immunogenicity studies in this cohort showed that 70% of vaccinated women maintained hemagglutination inhibition (HAI) antibody titers ≥1:40 to at least one vaccine strain until 8 months postvaccination [10]. There is controversy regarding the persistence of IIV3 protection in nonpregnant adults; recent studies reported persistence of HAI titers ≥1:40 and residual protection against infection in subsequent seasons without revaccination and that seroconversion to IIV3 was lower in adults who received IIV3 in the previous year compared to those who were vaccinated for the first time [11–13]. The duration of protection following immunization during pregnancy has not been studied until now [14].

HAI titers ≥1:40 are putative correlates for 50% protection against influenza disease in healthy young adults [15]. Immune responses to IIV3 occur within 2 weeks postvaccination, with maximum levels reached 4–6 weeks postvaccination [16,17]. While HAI titers decline with time, the clinical implication of the decay for protection against influenza illness in the subsequent influenza season has not been well characterized [13]. Adaptations of the maternal immune system, such as pregnancy-induced suppression of cellular immunity to enable the accommodation of the allogenic fetus, may influence the immunogenicity of influenza vaccines [18,19]. Despite this immune modulation, studies have reported similar rates of seroconversion and seroprotection in pregnant and nonpregnant women after vaccination [9,19–23].

The goals of this study were to evaluate the persistence of HAI antibodies to the three influenza strains included in IIV3 administered to pregnant women during the previous influenza season and to determine if the efficacy of IIV3 against PCR-CI infection persists in the subsequent season.

## 2. Methods

### 2.1. Study design

**Figure 1.**
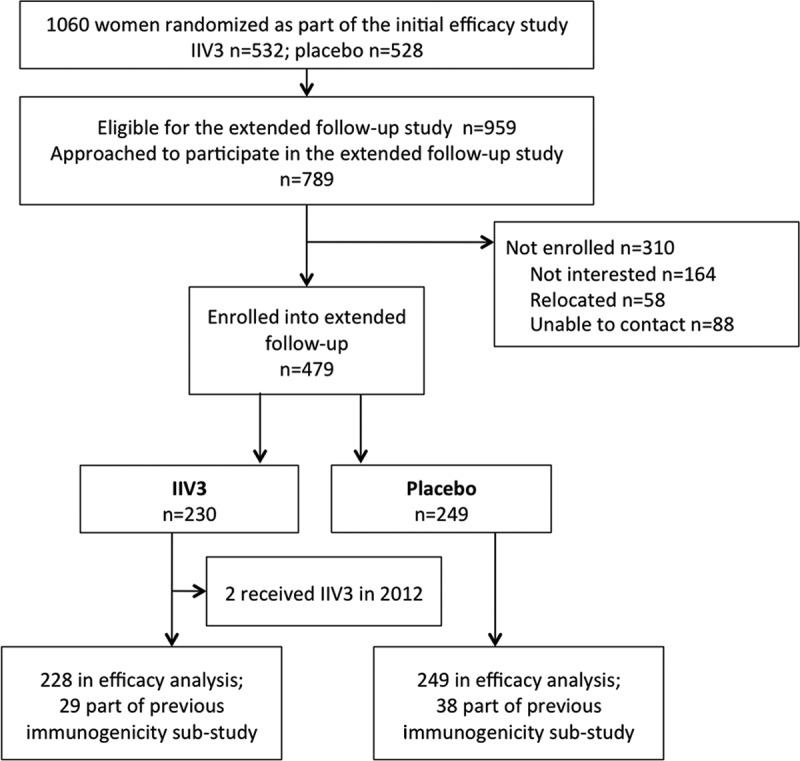
Study allocation and participants. IIV3, inactivated trivalent influenza vaccine

Between March and October 2012, HIV-uninfected women who completed a double-blind, randomized, placebo-controlled trial assessing the safety, immunogenicity and efficacy of IIV3 in pregnant women in 2011 were invited to participate in the extended follow-up study (Supplementary Figure 1). In the initial trial, pregnant women from Soweto, South Africa, were randomized to receive either a single dose of IIV3 or placebo between 20 and 36 weeks of gestation. The primary analyses of the vaccine efficacy (VE) and immunogenicity in the mothers and their infants up to 24 weeks postdelivery have been reported [9,10]. Women eligible to participate in the extended follow-up study were those who completed the initial 2011 IIV3 trial and had not received additional influenza vaccination outside of the 2011 trial. Recommended influenza vaccine formulation by the World Health Organization in 2011 and 2012 remained identical, providing an opportunity to investigate the duration of protection after seasonal influenza vaccination in the previous season [24,25]. Figure 1 depicts the overall study design. Details regarding the initial study design and enrolment have been described earlier [9].

Blood samples (5–10 mL) from all participants were collected at enrolment into the extended follow-up study for evaluation of HAI titers to the influenza strains contained in the IIV3 formulation used, i.e. A/California/7/2009 (A/H1N1pdm09), A/Victoria/210/2009 (A/H3N2), and B/Brisbane/60/2008-like virus (B/Victoria lineage). Persistence of antibodies to the three strains was measured by HAI assay on plasma as described in [9].

Active surveillance for clinical illness was conducted by sending weekly short message service reminders of influenza illness symptoms and a request to attend the study clinic at Chris Hani Baragwanath Academic Hospital should the participant have pre-specified signs suggestive of influenza-like illness (ILI). As during the main trial in 2011, physical examination and screening for symptoms was performed on all participants attending the study clinic during the course of the study period. Nasopharyngeal and oro-pharyngeal swabs were collected in viral transport media at the time of illness visits. Specimens were tested using multiplex reverse transcription real-time PCR assays for influenza A and B as previously described [9]. In order to check for loss to follow-up and missed illness episodes, participants were contacted at the end of the follow-up period by phone.

### 2.2. Statistical analysis

A convenience sample was enrolled (Figure 1). Demographic characteristics were compared between IIV3 and placebo groups using Chi-square test or Fisher’s exact-test for proportions and the Student’s *t*-test or Mann–Whitney *U*-test for continuous variables. In the main immunogenicity analyses, participants with an episode of PCR-CI during the 2011 IIV3 study were excluded from the respective strain analyses. Geometric mean titers (GMT) for each vaccine strain were estimated using logarithmic transformation of HAI titers and compared between study groups by the Student’s *t*-test. Despite HAI titers showing skewed distribution, as frequently observed in immune response data, normal parametric methods are found to be appropriate when sample sizes are large (*n* > 100) [26]. Seroprotection rates as the proportion of women with HAI titer ≥1:40 were compared between study groups by Chi-square test. Logistic regression was used to assess predictors of seroprotection. For participants that were included in the 2011 nested immunogenicity substudy and had bloods collected at prevaccination, 1-month postvaccination, at delivery, and at 24 weeks postdelivery, fold-decline in GMT and seroprotection rates over time were assessed. Time between vaccination and enrolment into the extended follow-up study was stratified in three time periods: 6–10 months, 11–15 months, and 16–20 months, and GMT were compared by one-way ANOVA.

All events occurring between signing informed consent and December 2012 were included in the efficacy analyses. Subanalyses were undertaken including only the PCR-CI cases detected during the period with influenza viral activity in the study area, and only the women enrolled before the start of the influenza season (Supplementary Figure 1) [27]. VE was estimated as 1 minus the incidence rate ratio between IIV3- and placebo-recipients. Cases were specified as women with PCR-CI and other endpoints for estimation of VE included combined PCR-CI for 2011 and 2012, protocol defined ILI and at least one illness visit. To control for differences in time since vaccination, incidence rates were calculated as density incidence using Poisson regression. Time to event data was right censored after the first episode of the outcome of interest or at study termination. Survival analysis evaluated between group differences in time to first influenza episode using Kaplan–Meier curves and the log-rank test. Data were collected using Research Electronic Data Capture [28] and analyzed using STATA version 13.1 (College Station, TX, U.S.A.). *p*-Values of <0.05 were considered to have statistical significance; all tests were two-sided.

### 2.3. Ethics

All participants provided written informed consent prior to study initiation. Approval was obtained from the Human Research Ethics Committee of the University of the Witwatersrand (approval number 120107), and the trial was conducted in accordance with Good Clinical Practice guidelines. The initial trial was registered at ClinicalTrial.gov (NCT01306669).

## 3. Results

Of the 959 women eligible for the extended follow-up, 479 agreed to participate, including 230 IIV3- and 249 placebo-recipients in 2011. Maternal age, gestational age at vaccination, and percentage of primigravid women were similar between the women who enrolled and those who were eligible but did not enroll in the extended follow-up study (Supplementary Table 1). Two IIV3-recipients were excluded from the analyses due to receipt of IIV3 in 2011 and 2012. At enrolment into the extended follow-up study, the average age was 27.2 years (standard deviation [S.D.] = 5.2) and the mean time since vaccination in 2011 was 372 days (S.D. = 65). There were no differences between IIV3- and placebo-recipients in baseline characteristics (Table 1). Average time of follow-up between vaccination and study conclusion was 592 (S.D. = 53) days. Three participants had no HAI results available and were not included in the immunogenicity analyses (1 IIV3- and 2 placebo-recipients).

**Table 1.** Characteristics at enrolment into the extended follow-up study and follow-up time.

	Total	IIV3	Placebo
Total number of women	477	228	249
Mean age (years)	27.2 ± 5.2	27.6 ± 5.2	26.9 ± 5.3
Mean body mass index (kg/m^2^)	28.2 ± 6.1	28.1 ± 5.7	28.2 ± 6.4
Mean gestational age at vaccination (weeks)	27.3 ± 4.2	27.3 ± 4.2	27.3 ± 4.2
Primigravid [no. (%)]	158 (33.1)	66 (29.0)	92 (37.0)
Current use of medication^a^ [no. (%)]	15 (3.1%)	8 (3.5%)	7 (2.8%)
Mean time since vaccination in 2011 (days)	372 ± 65	370 ± 64	374 ± 65
Mean time since delivery (days)	292 ± 74	291 ± 73	292 ± 74
Mean time of follow-up (days)	592 ± 53	593 ± 51	590 ± 54
Women enrolled before the start of 2012 influenza season^b^ [no. (%)]	239 (50.1%)	123 (53.9%)	116 (46.6%)

Plus and minus values are means ± standard deviation (S.D.). IIV3, inactivated trivalent influenza vaccine.

^a^Examples of current use of medication at the time of enrolment into extended follow-up study included nifedipine, perindopril, hydrochlorothiazide, furosemide, spironolacton, albuterol, aspirin, simvastatin, carbamazepine, and antihistamine.

^b^The influenza season started on 21 May 2012.

Of the 474 participants included in the immunogenicity analysis, 29 IIV3-recipients and 38 placebo-recipients participated in the immunogenicity substudy in 2011 and had HAI titers evaluated at four time-points [10]. Among IIV3-recipients who participated in the immunogenicity substudy in 2011, there was a threefold decline in GMT for A/H3N2, fourfold for A/H1N1pdm09, and fivefold for B/Victoria from 1-month post-IIV3 until enrolment in the extended follow-up (Table 2).

**Table 2.** Trivalent inactivated influenza vaccine immunogenicity in women who were part of the 2011 immunogenicity substudy.

	IIV3		Placebo		*p*-Value	
Time	GMT (95% CI)	%SP (*n*)	GMT (95% CI)	%SP (*n*)	GMT	%SP
*t* = 1 (1-month postvaccination)^a^						
A/H3N2	163.9	96.6	16.4	26.3	<0.001	<0.001
(*n* = 67)	(113.3–237.1)	(28)	(12.0–22.3)	(10)		
A/H1N1	258.1	96.6	45.4	57.9	<0.001	<0.001
(*n* = 67)	(173.1–384.7)	(28)	(27.2–75.9)	(22)		
B/Victoria	252.0	100	23.7	43.2	<0.001	<0.001
(*n* = 66)	(169.9–373.7)	(29)	(17.9–31.3)	(16)		
*t* = 2 (enrolment into extended follow-up)						
A/H3N2	57.2	75.9	12.7	18.4	<0.001	<0.001
(*n* = 67)	(36.7–89.2)	(22)	(9.6–16.7)	(7)		
A/H1N1	63.0	72.4	23.6	42.1	0.004	0.013
(*n* = 67)	(40.5–98.0)	(21)	(14.7–37.7)	(16)		
B/Victoria	48.4	62.1	11.8	16.2	<0.001	<0.001
(*n* = 66)	(30.7–76.4)	(18)	(8.9–15.7)	(6)		
All women enrolled into the extended follow-up study, excluding PCR-CI cases^b^						
A/H3N2	46.3	65.2	13.4	24.4 (59)	<0.001	<0.001
(*n* = 469)	(38.9–55.1)	(148)	(11.8–15.1)			
A/H1N1	44.7	61.4	17.2	30.5 (75)	<0.001	<0.001
(*n* = 469)	(37.4–53.4)	(137)	(14.8–20.0)			
B/Victoria	42.2	63.0	11.3	11.4 (28)	<0.001	<0.001
(*n* = 473)	(36.9–48.3)	(143)	(10.3–12.5)			

*p*-Value GMTs derived from *t*-test on log 10 transformed HAI titers. *p*-Value %SP derived from Chi-square test or Fisher’s exact test when appropriate. IIV3, inactivated trivalent influenza vaccine; GMT, geometric mean titer; SP, seroprotection defined as HAI ≥1:40; PCR-CI, polymerase-chain reaction-confirmed influenza in 2011. ^a^Between vaccination and *t* = 1, one participant who had a PCR-CI event in 2011 was excluded for the corresponding strain analysis: B/Victoria. At *t* = 1, only women at 28–35 days postvaccination are included.

^b^Overall, 11 participants who had a PCR-CI event in 2011 were excluded for the corresponding strain analysis: 5 for A/H3N2, 5 for A/H1N1, and 1 for B/Victoria.

IIV3-recipients had a threefold or higher GMT for all vaccine strains compared to placebo-recipients at enrolment into the extended study. The proportion of women with HAI titers ≥1:40 at enrolment into the extended follow-up study among the IIV3 and placebo groups was 65.2% vs. 24.4% for A/H3N2, 61.4% vs. 30.5% for A/H1N1pdm09, and 63.0% vs. 11.4% for B/Victoria (*p* < 0.001 for all comparisons) (Table 2).

**Figure 2.**
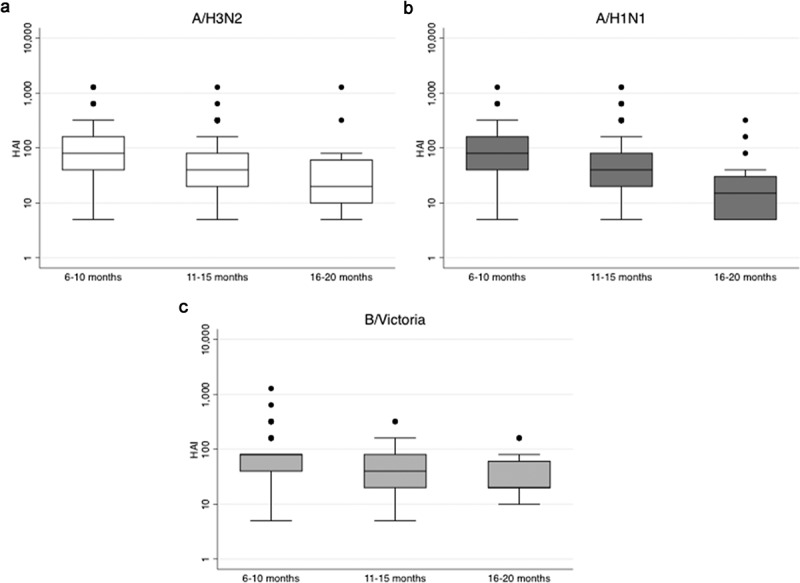
Box-plots of HAI titers at enrolment into the extended follow-up study by months since vaccination. A) A/H3N2 HAI titers; B) A/H1N1 HAI titers; C) B/Victoria HAI titers.*P*-values comparing time since vaccination between groups (6–10 months vs. 11–15 months vs. 16–20 months) in IIV3-recipients derived from one way ANOVA on log10 transformed HAI titers; *p*-values <0.01 for all comparisons.The y-axis has been log scaled.HAI, haemagglutination inhibition; IIV3, inactivated trivalent influenza vaccine.

Time between receipt of IIV3 and blood collection at enrolment into the extended follow-up study was stratified in three time periods (6–10 months, 11–15 months, and 16–20 months). GMT in IIV3-recipients were significantly lower at 11–15 and 16–20 months postvaccination compared to 6–10 months postvaccination (Figure 2). An analysis of predictors of HAI ≥1:40 1 year after initial vaccination showed that women ≥25 years of age were less likely to maintain protective titers to A/H3N2, A/H1N1pdm09, to ≥2 influenza vaccine strains or to all three strains, compared with those <25 years of age. Women were also less likely to have HAI titers ≥1:40 when time since vaccination was 11–15 months and 16–20 months, compared to 6–10 months (Table 3). Other characteristics, such as body mass index, gestational age at vaccination, time since delivery, gravidity, and use of medication at time of enrolment into extended follow-up, did not influence the proportion with seroprotective titers.

**Table 3.** Predictors of seroprotection in IIV3-recipients at enrolment into the extended follow-up.

Predictor	Nonseroprotected *n*/*N* (%)	Seroprotected *n*/*N* (%)	OR (95% CI)	*p*-Value
Age at vaccination, by strain				
A/H3N2	15/79 (19.0%)	65/148 (43.9%)	Referent	<0.001
<25 Years	64/79 (81.0%)	83/148 (56.1%)	0.30 (0.16–0.57)	
≥25 Years				
A/H1n1pdm09	21/86 (24.4%)	58/137 (42.3%)	Referent	0.007
<25 Years	65/86 (75.6%)	79/137 (57.7%)	0.44 (0.24–0.80)	
≥25 Years				
B/Victoria	25/84 (29.8%)	55/143 (38.5%)	Referent	0.19
<25 Years	59/84 (70.2%)	88/143 (61.5%)	0.68 (0.38–1.21)	
≥25 Years				
≥1 Strain	4/19 (21.1%)	76/208 (36.5%)	Referent	0.19
<25 Years	15/19 (79.0%)	132/208 (63.5%)	0.46 (0.15–1.45)	
≥25 Years				
≥2 Strains	16/79 (20.3%)	64/148 (43.2%)	Referent	0.001
<25 Years	63/79 (79.8%)	84/148 (56.8%)	0.33 (0.18–0.63)	
≥25 Years				
Three strains	41/151 (27.2%)	39/76 (51.3%)	Referent	<0.001
<25 Years	110/151 (72.9%)	37/76 (48.7%)	0.35 (0.20–0.63)	
≥25 Years				
Time since vaccination, by strain				
A/H3N2	15/79 (19.0%)	54/148 (36.5%)	Referent	0.04
6–10 Months	54/79 (68.4%)	88/148 (59.5%)	0.48 (0.24–0.96)	0.002
11–15 Months	10/79 (12.7%)	6/148 (4.1%)	0.16 (0.05–0.52)	
16–20 Months				
A/H1N1pdm09	17/86 (19.8%)	52/137 (38.0%)	Referent	0.03
6–10 Months	57/86 (66.3%)	81/137 (59.1%)	0.49 (0.25–0.93)	0.001
11–15 Months	12/86 (14.0%)	4/137 (2.9%)	0.11 (0.03–0.38)	
16–20 Months				
B/Victoria	16/84 (19.1%)	53/143 (37.1%)	Referent	0.01
6–10 Months	59/84 (70.2%)	83/143 (58.0%)	0.43 (0.23–0.83)	0.01
11–15 Months	9/84 (10.7%)	7/143 (4.9%)	0.24 (0.08–0.74)	
16–20 Months				
≥1 Strain	1/19 (5.3%)	68/208 (32.7%)	Referent	0.06
6–10 Months	14/19 (73.7%)	128/208 (61.5%)	0.13 (0.02–1.05)	0.004
11–15 Months	4/19 (21.0%)	12/208 (5.8%)	0.03 (0.00–0.35)	
16–20 Months				
≥2 Strains	12/79 (15.2%)	57/148 (38.5%)	Referent	0.003
6–10 Months	56/79 (70.9%)	86/148 (58.1%)	0.34 (0.17–0.70)	<0.001
11–15 Months	11/79 (13.9%)	5/148 (3.4%)	0.09 (0.03–0.32)	
16–20 Months				
Three strains	35/151 (23.2%)	34/76 (44.7%)	Referent	0.01
6–10 Months	100/151 (66.2%)	42/76 (55.3%)	0.46 (0.25–0.85)	0.07
11–15 Months	16/151 (10.6%)	0/76 (0%)	1	
16–20 Months				

Variables that were found not to be significantly associated with seroprotection include body mass index, gestational age at vaccination, gravidity, time since delivery and use of medication at the time of enrolment of the extended follow-up. IIV3, inactivated trivalent influenza vaccine; CI, confidence interval; OR, odds ratio.

**Figure 3.**
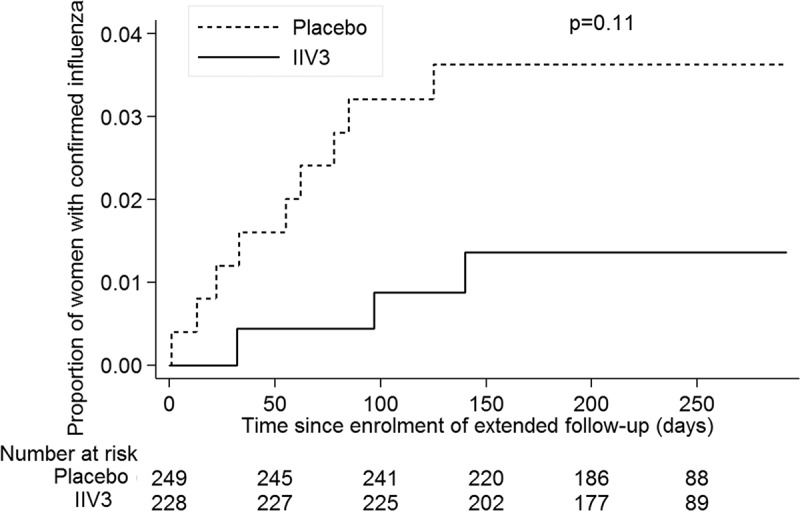
Kaplan-Meier estimates of the proportion of influenza confirmed cases in 2012 according to intervention group. IIV3, inactivated trivalent influenza vaccine

During 2012, 12 participants had an episode of PCR-CI, including 3 in the IIV3-group (mean time post-IIV3: 417 days ± 25) and 9 in the placebo-group. The incidence was lower among IIV3-recipients (0.68 [95% confidence interval [95% CI]: 0.22, 2.10] per 1000 person/months) compared to placebo-recipients (1.87 [95% CI: 0.97, 3.59] per 1000 person/months), with a VE of 63.80% (95% CI: −33.71%, 90.20%; *p* = 0.13) (Table 4). The corresponding Kaplan–Meier curve is presented in Figure 3. When combining PCR-CI episodes of 2011 and 2012, 25 women had a PCR-CI episode: 7 in the IIV3-group and 18 in the placebo-group, yielding a VE of 58.31% (95% CI: 0.19%, 82.59%; *p* = 0.05) (Table 4). One woman had a PCR-CI in both 2011 and 2012. The predominant strain of influenza detected in the 2012 symptomatic cases was A/H3N2 (*N* = 1 in IIV3-group and *N* = 5 in placebo-group). The VE estimates were similar when the three cases (*N* = 1 in the IIV3-group and *N* = 2 in the placebo-group) of symptomatic B/Yamagata infections, which was not part of the IIV3 formulation, were excluded from the analysis (Table 4). The VE estimate over the entire year of 2012 was similar to the VE restricted to the time when influenza circulated in 2012 and to the estimate when limiting the analysis to women enrolled before the start of the influenza season (Table 4). The incidence rates of ILI and illness visits were similar between IIV3- and placebo-recipients (Table 4).

**Table 4.** Incidence rates and vaccine efficacy (VE) of IIV3 vaccination in preventing PCR-confirmed influenza illness.

	IIV3 *n* = 228		Placebo *n* = 249			
Outcome	*N*	Rate (95% CI)^a^	*N*	Rate (95% CI)^a^	VE (95% CI)	*p*-Value
PCR-confirmed influenza in 2012^b^						
With inclusion of B/Yamagata	3	0.68	9	1.87	63.80	0.13
		(0.22, 2.10)		(0.97, 3.59)	(−33.71, 90.20)	
With exclusion of B/Yamagata	2	0.45	7	1.46	69.00	0.14
		(0.11, 1.81)		(0.70, 3.07)	(−49.25, 93.56)	
H3N2 detection	1	0.23	5	1.04	78.28	0.16
		(0.03, 1.60)		(0.43, 2.49)	(−85.90, 97.46)	
PCR-confirmed influenza restricted to influenza season^C^						
With inclusion of B/Yamagata	3	0.68	8	1.67	59.39	0.18
		(0.22, 2.10)		(0.83, 3.33)	(−53.05, 89.23)	
With exclusion of B/Yamagata	2	0.45	6	1.26	64.00	0.21
		(0.11, 1.81)		(0.56, 2.80)	(−78.36, 92.73)	
PCR-confirmed influenza in 2011 and 2012 combined^d^						
With inclusion of B/Yamagata	7	1.60	18	3.85	58.31	0.05
		(0.77, 3.37)		(2.43, 6.11)	(0.19, 82.59)	
With exclusion of B/Yamagata	6	1.38	13	2.81	50.80	0.15
		(0.62, 3.07)		(1.63, 4.83)	(−29.44, 81.30)	
PCR-confirmed influenza restricted to women enrolled before the start of the 2012 influenza season	1	0.41	4	1.78	76.73	0.19
		(0.06, 2.93)		(0.67, 4.73)	(−108.22, 97.40)	
First episode of influenza-like illness^e^	23	5.18	28	5.81	10.80	0.69
		(3.45, 7.80)		(4.01, 8.42)	(−54.85, 48.61)	
At least one illness visit	125	28.18	131	27.19	−3.62	0.78
		(23.65, 33.58)		(22.91, 32.27)	(−32.40,18.90)	

IIV3, inactivated trivalent influenza vaccine; PCR, polymerase-chain reaction.

^a^Incidence rates were calculated as number of cases per 1000 person/months, using person-time between vaccination and event or end of study.

^b^Among IIV3-recipients and placebo-recipients, PCR-confirmed B/Victoria was identified in one and two women, respectively, B/Yamagata in one and one women, B/Yamagata and B/Victoria co-infection in one woman in the placebo group, no A/H1N1pdm09 infections occurred.

^c^Analysis restricted to the influenza season, between 21 May 2012 and 14 October 2012.

^d^In 2011, among IIV3-recipients and placebo-recipients, PCR-confirmed A/H1N1 was identified in four and one women, respectively, A/H3N2 in zero and five women, respectively, B/Yamagata in zero and three women, respectively, and B/Victoria in one woman in the placebo group. One woman had PCR-CI in both 2011 and 2012.

^e^First episode of influenza-like illness was defined as presence of fever (≥38°C on oral measurement) or chills/rigors, or feeling feverish in past for <7 days and any of cough/sore throat/pharyngitis, or any of muscle ache/joint ache/headache, or any of feeling shortness of breath/difficulty breathing/chest pain while breathing.

Among IIV3-recipients, one participant had a PCR-CI episode of A/H3N2 in 2012 and one of B/Victoria; their HAI titers for the corresponding strain at enrolment into the extended follow-up were 1:5 and 1:20 respectively. Among the seven placebo-recipients with a PCR-CI episode by a vaccine strain, one (11.1%) had HAI titers 1:40 for the detected strain (A/H3N2) and in the other cases none had HAI titers ≥1:40 for the corresponding strain at enrolment into the extended follow-up.

## 4. Discussion

At an average of 372 days postvaccination, IIV3-recipients had significantly higher GMT and prevalence of seroprotective titers compared to placebo-recipients. Influenza infection attack rate was lower in the IIV3-group than in the placebo-group during the second influenza season, with a VE point estimate of 63.8%. The crude attack rates (number of cases divided by total number of participants) during the extended follow-up in 2012 were the same as first season attack rates in 2011: 1.3 in IIV3- and 3.6 in placebo-recipients [9]. However, due to the smaller number of participants in 2012 compared with 2011, the extended follow-up study did not have sufficient power to confirm a VE of this magnitude (25% power to detect a 60% reduction). The combined results for 2011 and 2012 showed a significant VE point estimate of 58.3%.

Women who received IIV3 during pregnancy, had detectable HAI titers against the influenza vaccine strains approximately 12 months after vaccination, with 61–65% of women maintaining HAI titers ≥1:40. Our findings are consistent with the literature that reported persistence of antibodies 1 year after IIV3 vaccination of nonpregnant adults [29–31]. Moreover, other studies showed that high-risk populations, such as the elderly and lung transplants patients, had persistent HAI titers above seroprotective levels in the subsequent season [32,33]. The proportion of seroprotected women in our study was lower than the number of participants reported by Petrie et al. with HAI titers ≥32 (89.1% for A/H3N2 and 95.6% for influenza B) 18 months following vaccination [13]. Nevertheless, our data show that vaccination during pregnancy, despite the pregnancy related immune suppression, results in a high proportion of women with seroprotective titers in the second influenza season, and this was associated with younger age and shorter time since vaccination. Although in our previous analysis younger age was not found to be associated with better response to IIV3 in HIV-uninfected women [10]; a higher proportion of women <25 years maintained HAI ≥1:40 1 year after initial vaccination compared to older women. Since the half-life of the antibodies induced by vaccination in these women is approximately 106–121 days [10], it is not surprising that titers were lower at 11–20 months postvaccination compared to 6–10 months postvaccination, nonetheless this observation has implications on when would be the best time to re-vaccinate with seasonal influenza vaccine.

Results from previous trials suggested that protection against influenza infection after vaccination might persist for more than 12 months [11–13]. Our VE estimate for PCR-CI was similar to that in the original IIV3 study conducted in women up to 24 weeks postpartum in South Africa in 2011 (50.4%, 95% CI: 14.5%, 71.2%) [9] and to the VE reported after vaccination during first season in pregnant women in the United States (76%, 95% CI 22%, 92%) [34].

The 2012 influenza season in South Africa was characterized by the bi-phasic co-circulation of influenza A/H3N2 (57–61%) and influenza B (39–41%, subdivided in B/Victoria 68%, B/Yamagata 32%), with minimal A/H1N1pdm09 (1%) [35–37]. Phylogenetic analysis showed that the 2012 A/H1N1pdm09 and A/H3N2 circulating strains were similar to the circulating strains in 2011 [9] and that the majority of B/Victoria/lineage-like viruses were B/Brisbane/60/2008-like [36], therefore, sustained second-season efficacy may be explained by conservation of circulating virus strains.

We did not detect clinical efficacy of influenza vaccination against ILI or medically attended illness visits, which was similar to what was reported in the United States cohort [38]. In contrast, a study in Bangladesh found reduced rates of respiratory illness with fever and fewer clinic visits in mothers receiving influenza vaccine during pregnancy when evaluated during the first influenza season [39]. These discrepancies may be due to differences in respiratory viruses circulating in Bangladesh and South Africa and the United States. Of note, influenza infections in Bangladesh are not seasonal.

Strengths of our study include minimal risk of bias due to the randomized and blinded nature of the parent study; similar demographic characteristics of the women who were enrolled in the extended follow-up compared with those who did not; use of PCR-CI as outcome measure; and the duration of the follow-up. Because of the location and timing of the trial, particularly the fact that the same influenza strains circulated in both years and they were well matched to the vaccine strains with the exception of B/Yamagata, we could investigate sustained protection following vaccination through two consecutive influenza seasons.

This study was limited by the single detection of HAI titers at enrolment into the extended follow-up. For more insight in the durability of the antibody response, it would have been preferable to obtain immunogenicity data at subsequent time points. Noticeably, our results are limited to HIV-uninfected population and cannot be extrapolated to HIV-infected women. Moreover, the women were vaccine naïve at enrolment into the initial trial, and the results may be different in women who are vaccinated every year.

In conclusion, protective effects of influenza vaccination during pregnancy were observed during two successive influenza seasons with similar circulating strains, which were well matched to the vaccine formulation. IIV3 was shown to provide a sustained immune response, including persistence of seroprotective antibodies up to 12 months, and a trend indicating long-term efficacy against influenza infection up to 19 months against antigenically similar strains.

## Key issues

- IIV3 is recommended for pregnant women.
- Limited data are available regarding duration of protection after vaccination.
- Women who participated in a randomized placebo-controlled trial of IIV3 in 2011 were enrolled in this study in 2012, taking advantage that the IIV3 formulation did not change between 2011 and 2012.
- At study entry, the HAI geometric mean titers in IIV3-recipients were ≥3-fold higher than in placebo-recipients.
- The proportion of women with HAI titers ≥1:40 was significantly greater in vaccinees (63%) compared to placebo-recipients (22%; p < 0.001).
- In 2012, 3 IIV3-recipients and 9 placebo-recipients had PCR-confirmed influenza illness, corresponding to a VE of 63.8% (95% confidence interval [95%CI]: −33.7%, 90.2%).
- The combined VE for 2011 and 2012 was 58.3% (95%CI: 0.2%, 82.6).
- One year after vaccination, the majority of women who received IIV3 during pregnancy had HAI titers above the putative threshold for protection against influenza illness.
- In the second-influenza season, a trend towards protection against influenza disease was shown.

## Supplementary Material

Supplementary_Material.zip
