# Supplementary material for: Influenza vaccination of pregnant women protects them over two consecutive influenza seasons in a randomized controlled trial
Source: Expert Rev Vaccines. 2016 Jun 6;15(8):1055–62. doi: 10.1080/14760584.2016.1192473 (PMC4950453; doi:10.1080/14760584.2016.1192473)
Supplement: Supplementary_Material.zip [file ierv_a_1192473_sm6848.zip › Suppfigure_11april.docx]

**Supplementary figure 1. Enrolment into the extended follow-up study over time in 2012**

Information on Influenza season obtained from:

Influenza surveillance report - South Africa. Available at: <http://www.nicd.ac.za/?page=seasonal_influenza&id=72>
