# Supplementary material for: Influenza vaccination of pregnant women protects them over two consecutive influenza seasons in a randomized controlled trial
Source: Expert Rev Vaccines. 2016 Jun 6;15(8):1055–62. doi: 10.1080/14760584.2016.1192473 (PMC4950453; doi:10.1080/14760584.2016.1192473)
Supplement: Supplementary_Material.zip [file ierv_a_1192473_sm6848.zip › Supptable_11apr.docx]

**Supplementary Table 1. Characteristics of women enrolled into the extended follow-up study and women eligible but not enrolled**

|  | **Total** | **Enrolled** | **Eligible but not enrolled** |
| --- | --- | --- | --- |
| Total no. of women | 959 | 479 | 480 |
| Mean age in years at 1 January 2012 | 26.9 ± 5.3 | 26.9 ± 5.3 | 26.9 ± 5.3 |
| Mean gestational age at time of vaccination, weeks | 27.2 ± 4.3 | 27.3 ± 4.2 | 27.0 ± 4.4 |
| Primigravid, no. (%) | 313 (32.6) | 158 (33.0) | 155 (32.3) |

Plus and minus values are means ± standard deviation (SD).
